# Supplementary material for: Is Alexithymia a Trait or a State? Temporal Stability in a Three-Wave Longitudinal Study
Source: J Clin Med. 2025 Apr 11;14(8):2628. doi: 10.3390/jcm14082628 (PMC12027918; doi:10.3390/jcm14082628)
Supplement: Supplementary file 1 [file jcm-14-02628-s001.zip › jcm-3573341-supplementary.pdf]

Supplementary Table S1. Pearson correlations between the study variables (*n* = 73).

Variables	1	2	3	4	5	6	7	8	9	10	11	12	13	14	15
1. PAQ-S T1	—														
2. PAQ-S T2	0.67 ***	—													
3. PAQ-S T3	0.58 ***	0.56 ***	—												
4. PHQ-4 Anxiety T1	0.30 **	0.26 *	0.22	—											
5. PHQ-4 Anxiety T2	0.18	0.11	0.07	0.48 ***	—										
6. PHQ-4 Anxiety T3	0.21	0.14	0.24 *	0.47 ***	0.47 ***	—									
7. PHQ-4 Depression T1	0.36 **	0.23 *	0.25 *	0.70 ***	0.45 ***	0.40 ***	—								
8. PHQ-4 Depression T2	0.25 *	0.25 *	0.19	0.43 ***	0.76 ***	0.33 **	0.53 ***	—							
9. PHQ-4 Depression T3	0.22	0.29 *	0.28 *	0.31 **	0.38 ***	0.61 ***	0.34 **	0.54 ***	—						
10. GBB-8 T1	0.11	0.08	0.14	0.52 ***	0.33 **	0.30 **	0.48 ***	0.36 **	0.36 **	—					
11. GBB-8 T2	0.12	0.15	0.14	0.40 ***	0.56 ***	0.25 *	0.35 **	0.52 ***	0.30 **	0.75 ***	—				
12. GBB-8 T3	0.09	0.00	0.22	0.22	0.31 **	0.34 **	0.22	0.17	0.38 **	0.56 ***	0.61 ***	—			
13. WHO-5 T1	-0.19	-0.16	-0.16	-0.73 ***	-0.50 ***	-0.43 ***	-0.67 ***	-0.50 ***	-0.35 **	-0.50 ***	-0.42 ***	-0.24 *	—		
14. WHO-5 T2	-0.28 *	-0.23 *	-0.22	-0.58 ***	-0.69 ***	-0.39 ***	-0.57 ***	-0.72 ***	-0.48 ***	-0.53 ***	-0.64 ***	-0.43 ***	0.68 ***	—	
15. WHO-5 T3	-0.22	-0.05	-0.34 **	-0.33 **	-0.34 **	-0.54 ***	-0.42 ***	-0.35 **	-0.56 ***	-0.42 ***	-0.33 **	-0.57 ***	0.49 ***	0.56 ***	—

Note. * $p < 0.05$; ** $p < 0.01$; *** $p < 0.001$.

Supplementary Table S2. Multiple regression analysis parameters ($n = 73$).

Models	Predictors		Sum of Squares	df	Mean Square	F	p
Step 1	3 predictors (T1, T2, and T3 of GBB-8)	Regression	205.09	3	68.36	1.14	0.341
		Residual	4150.99	69	60.16		
		Total	4356.08	72			
Step 2	6 predictors (T1, T2, and T3 of GBB-8 and PHQ-4 Depression)	Regression	534.39	6	89.07	1.54	0.180
		Residual	3821.69	66	57.90		
		Total	4356.08	72			
Step 3	9 predictors (T1, T2, and T3 of GBB-8, PHQ-4 Depression, and PHQ-4 Anxiety)	Regression	758.86	9	84.32	1.48	0.176
		Residual	3597.22	63	57.10		
		Total	4356.08	72			
Step 4	12 predictors (T1, T2, and T3 of GBB-8, PHQ-4 Depression, PHQ-4 Anxiety, and WHO-5)	Regression	887.52	12	73.96	1.28	0.254
		Residual	3468.56	60	57.81		
		Total	4356.08	72			
Step 5	14 predictors (T1, T2, and T3 of GBB-8, PHQ-4 Depression, PHQ-4 Anxiety, and WHO-5 as well as T1 and T2 of PAQ-S)	Regression	2124.56	14	151.75	3.94	< 0.001
		Residual	2231.52	58	38.47		
		Total	4356.08	72			

Supplementary Table S3. Changes of *F*-values and R^2-values between different steps of the multiple regression analysis ($n = 73$).

Models	Predictors	R^2	Adjusted R^2	R^2 Change	*F* Change	df1	df2	*p*
Step 0	—	0.00	0.00	0.00	—	0	72	—
Step 1	3 predictors (T1, T2, and T3 of GBB-8)	0.05	0.01	0.05	1.14	3	69	0.341
Step 2	6 predictors (T1, T2, and T3 of GBB-8 and PHQ-4 Depression)	0.12	0.04	0.08	1.90	3	66	0.139
Step 3	9 predictors (T1, T2, and T3 of GBB-8, PHQ-4 Depression, and PHQ-4 Anxiety)	0.17	0.06	0.05	1.31	3	63	0.279
Step 4	12 predictors (T1, T2, and T3 of GBB-8, PHQ-4 Depression, PHQ-4 Anxiety, and WHO-5)	0.20	0.04	0.03	0.74	3	60	0.531
Step 5	14 predictors (T1, T2, and T3 of GBB-8, PHQ-4 Depression, PHQ-4 Anxiety, and WHO-5 as well as T1 and T2 of PAQ-S)	0.49	0.36	0.28	16.08	2	58	< 0.001

Supplementary Table S4. Multiple regression analysis coefficients, with the changes in alexithymia scores (ΔT1-T3 PAQ-S scores) as the dependent variable (*n* = 73).

Predictors	Unstandardized	Standard Error	Standardized	*t*	*p*	Tolerance
(Intercept)	1.39	0.93	–	1.49	0.140	–
ΔT1-T3 PHQ-4 Anxiety scores	0.02	0.61	0.01	0.04	0.970	0.64
ΔT1-T3 PHQ-4 Depression scores	0.36	0.50	0.10	0.74	0.465	0.71
ΔT1-T3 GBB-8 scores	0.04	0.15	0.04	0.30	0.768	0.78
ΔT1-T3 WHO-5 scores	-0.15	0.22	-0.10	-0.65	0.516	0.64

Note. $F(4,68) = 0.66$, $p = 0.625$, $R^2 = 4\%$, adjusted $R^2 = -2\%$.
